# Clinical Characteristics of Cerebral Venous Sinus Thrombosis in Patients with Systemic Lupus Erythematosus: A Single-Centre Experience in China

**DOI:** 10.1155/2015/540738

**Published:** 2015-05-19

**Authors:** Li Wang, Hua Chen, Yao Zhang, Wanli Liu, Wenjie Zheng, Xuan Zhang, Fengchun Zhang

**Affiliations:** ^1^Key Laboratory of Rheumatology and Clinical Immunology, Department of Rheumatology and Clinical Immunology, Peking Union Medical College Hospital, Chinese Academy of Medical Sciences, Ministry of Education, No. 1 Shuaifuyuan, Beijing 100730, China; ^2^Department of Neurology, Peking Union Medical College Hospital, Chinese Academy of Medical Sciences, No. 1 Shuaifuyuan, Beijing 100730, China; ^3^MOE Key Laboratory of Protein Science, School of Life Sciences, Tsinghua University, Beijing 100084, China

## Abstract

Clinical characteristics of systemic lupus erythematosus (SLE) patients complicated with cerebral venous sinus thrombosis (CVST) between 2000 and 2013 were analyzed through this retrospective, single-centre study. Of 4747 hospitalized SLE patients, 17 (0.36%, 12 females, average age 30) had CVST. Headache (88.2%) was the most common neurological symptom followed by nausea or vomiting (47.1%), conscious disturbance (41.2%), edema of eyelids or conjunctiva (35.3%), blurred vision or diplopia (35.3%), and seizure (35.3%). Increased intracranial pressure (ICP) occurred in 13 cases (76.5%). Magnetic resonance venography (MRV) detected thrombosis in the transverse (82.4%), sigmoid (52.9%), and sagittal (35.3%) sinuses, with frequent (70.6%) multiple sinus occlusions. Compared to SLE patients without CVST, SLE patients with CVST had a higher prevalence of thrombocytopenia and positive antiphospholipid antibodies and a higher SLE disease activity index (SLEDAI) score. 13 patients achieved improvement following glucocorticoids and immunosuppressants treatment, as well as anticoagulants, while 3 patients died at the hospital. CVST is relatively rare in SLE and tends to occur in active lupus patients. Intracranial hypertension is common but nonspecific clinical feature, so MRV evaluation is necessary to establish a diagnosis. Aggressive treatment for the rapid control of SLE activity combined with anticoagulants can improve the prognosis.

## 1. Introduction

Systemic lupus erythematosus (SLE) is a heterogeneous, multisystem autoimmune disease associated with the production of antibodies against self-antigens. Its pathologic basis is vasculitis induced by immune complex, which suggests that blood vessels might be an important target of autoimmunity overactivity. As a result, there is a wide variation in vessel involvement in SLE, including arteriostenosis and deep venous thrombosis, while glomerulonephritis and defused alveolar hemorrhage can also occur to a certain extent. Cerebral venous sinus thrombosis (CVST) is an uncommon disorder in SLE that is a manifestation of overlapping neuropsychiatric lupus (NPLE) [1] and vessel disease. CVST has been rarely reported and is potentially* fatal* to the patient due to the acute neurological deterioration that can occur. As such, practitioners should be familiar with this condition, so the correct diagnosis can be made as soon as possible to improve patient prognosis.

The etiology of CVST is complicated [2]. In SLE, the main culprits are vascular injuries caused by vasculitis. Antiphospholipid antibodies (APLs) and prothrombotic tendencies might also be responsible for CVST [3].

By investigating the clinical features of CVST in SLE and analyzing the differences between SLE patients with and without CVST, this study seeks to explore the characteristics and risk factors for CVST in SLE patients.

## 2. Patients and Methods

### 2.1. Patients

4747 SLE patients admitted to the Peking Union Medical College Hospital from January 2000 to December 2013 were reviewed. All patients were diagnosed as having SLE according to 1997 American College of Rheumatology (ACR) revised classification criteria [4]. In the SLE cohort, after exclusion of patients with infectious and traumatic CVST or incomplete data, we identified 17 cases (0.36%) complicated with CVST as indicated by clinical features, lumbar puncture, imaging materials (magnetic resonance venography (MRV)), and at least one neurologist's confirmation. For each case (*n* = 17), three age- and sex-matched controls (*n* = 51) were randomly selected from the contemporaneously admitted SLE patients without CVST. The local institutional review board approved the study. Because the study was based on a review of medical records that had been obtained for clinical purposes, the requirement for written informed consent was waived.

### 2.2. Statistical Analyses

SPSS version 16.0 (SPSS Inc., Chicago, USA) was used to statistically analyze the data. Numerical data and categorical data were expressed as mean ± SD (range) and percentage, respectively. The significance was estimated by Student's *t*-test, Pearson's chi-square, or Fisher's exact test (when expected frequencies were <5). *P* values < 0.05 were considered to be statistically significant.

## 3. Results

### 3.1. Demographic Characteristics

Among 17 SLE patients with CVST, 12 (70.6%) were women. The median age was 30 years old (range 12–52) and the SLE disease duration was 12 months (range 2–120). The mode of onset was acute (<48 h) in 12 patients (70.6%) and progressive (subacute or chronic) for the other 5 patients. In one case, CVST occurred as an initial manifestation. All patients came from North China.

### 3.2. Neurological Features of CVST in Lupus

Headache was the most common neurological manifestation (15/17, 88.2%), and among these cases, 8 patients suffered from nausea/vomiting (47.1%). Seven (41.2%) cases had conscious disturbance, 6 (35.3%) had edema of eyelids or conjunctiva, and 6 (35.3%) had blurred vision or diplopia. Hearing loss occurred in 2 cases (11.8%) while 6 cases had seizures (35.3%). As complications, subarachnoid hemorrhage (SAH, 4/17, 23.5%), sinusitis (3/17, 17.6%), and cerebral ischemia and infarction (2/17, 11.8%) were also found in SLE patients with CVST (Table 1).

**Figure 1:**
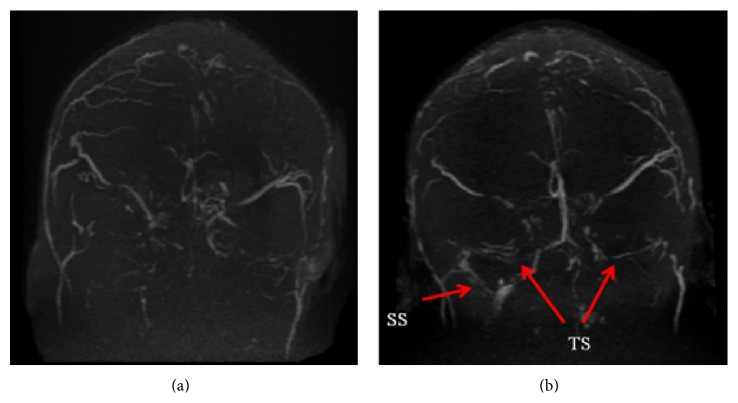
MRV of a SLE patient with CVST (Case number 5). (a)* Onset of CVST (June 2012)*. Occlusion of superior sagittal sinus (SSS), bilateral transverse sinus (TS), and bilateral sigmoid sinus (SS). (b)* Follow-up after treatment (November 2013)*. Recanalization of bilateral transverse sinus (TS) and right sigmoid sinus (SS) (arrows).

All patients underwent lumbar puncture, which showed that the intracranial pressure (ICP) was higher than 330 mm H_2_O in 7 patients (41.2%) and 6 patients' ICP ranged from 180 to 320 mm H_2_O, while the remaining 4 were lower than 180 mm H_2_O. Biochemical analysis of cerebrospinal fluid (CSF) indicated that the protein level was higher than normal in 9 patients (52.9%) and glucose and chloride levels were normal. Myelin basic protein (MBP) was elevated in 5 patients. From examination of MRV images, we found that thrombosis in the transverse sinus (TS) was the most common site (14/17, 82.4%), followed by sigmoid sinus (SS, 9/17, 52.9%). Thrombosis in the superior sagittal sinus (SSS, 6/17, 35.3%) and inferior sagittal sinus (ISS, 1/17, 5.9%) was also seen. Twelve patients suffered from more than one sinus thrombosis (12/17, 70.6%) (Figure 1(a)).

### 3.3. Systemic Manifestations of SLE

Most SLE patients with CVST (16/17, 94.1%) also had involvement of other organs, with lupus nephritis (12/17, 70.6%), hemocytopenia (10/17, 58.8%), arthritis (6/17, 35.3%), fever (6/17, 35.3%), rash (5/17, 29.4%), and serositis (5/17, 29.4%) being the most common conditions. The mean SLE disease activity index (SLEDAI) score (8 points of CVST excluded) of these patients was 12.1 ± 3.7. Twelve cases had hypoalbuminemia (12/17, 70.6%) and the mean serum albumin level of all 17 cases was (30.4 ± 7.5) g/L.

The prothrombin time and activated partial thromboplastin time were normal in most cases. Three patients had positive antiribosomal RNA-protein (rRNP) antibody. APLs, including anticardiolipin (5/17, 29.4%) antibodies, anti-*β*_2_GP_1_ antibodies (4/17, 23.5%), and lupus anticoagulants (6/17, 35.3%), were detected in 7 cases (7/17, 41.2%) in this cohort. Thrombophilia factors (protein S and protein C) were detected in 12 patients, and no protein C or S abnormality was found. Only one patient accompanied other thromboses in the veins (Table 2).

### 3.4. Comparison with SLE Cases without CVST

Compared with SLE patients without CVST, the incidence of thrombocytopenia (58.8% versus 23.5%, *P* = 0.007) and prevalence of APLs (41.2% versus 15.7%, *P* = 0.043) were significantly higher in SLE patients with CVST. SLE patients with CVST also had much higher disease activity (SLEDAI scores) (20.1 ± 3.7) when compared with SLE patients without CVST (12.8 ± 5.3, *P* = 0.001) (Table 3).

### 3.5. Treatments and Prognosis

All patients received glucocorticoid (GC) combined with immunosuppressant treatment. Thirteen patients were treated by pulse therapy with GC (methylprednisolone 0.5~1 g/d × 3~5 day). Cyclophosphamide (CTX) was selected as the preferred immunosuppressant for 17 patients, and FK506 or mycophenolate mofetil was added for 2 cases. Moreover, 1 patient had plasmapheresis.

Anticoagulation was utilized in 11 patients, with heparin or low molecular weight heparin (LMWH) being used with preference. Some patients did not receive anticoagulation because of severe SAH or thrombocytopenia. Seven patients had been intrathecally injected about 3 to 5 times with dexamethasone (10 mg), after which their CSF pressure decreased from over 330 mm H_2_O to 115~250 mm H_2_O.

Thirteen patients clinically improved, and one patient was lost to follow-up. Three patients died during hospitalization. Specifically, one patient died of cerebral hernia, the other one died of pneumonia, and the third one committed suicide. During the follow-up period (mean 36.8 ± 21.7 months), repeat of cerebral MRV was performed in 8 patients and 5 of them had partial or total recanalization of CVST (Figure 1(b)). No patient experienced a relapse of CVST.

## 4. Discussion

NPLE is one of the most important manifestations of SLE, which occurs in 14%~80% of adult [5] and 22%~95% of pediatric [6, 7] SLE patients. In 1999, ACR named NPLE into 19 categories [8]. CVST is one of the rare and severe cerebrovascular diseases, and its clinical features lack specificity. Thus, rheumatologists should be aware of this rare but potentially fatal complication of SLE.

As an uncommon disorder that accounts for about 0.5% of all strokes [9], CVST presents as a variety of neurologic symptoms and can lead to severe morbidity and mortality [10]. The causes of CVST include infection, dehydration, local trauma, rheumatologic diseases such as SLE and Behçet's disease (BD) [11] as well as thrombophilia including nephritic syndrome, antithrombin III deficiency, pregnancy, cancer, and use of oral contraceptives [2]. The current study showed that the incidence of CVST was about 0.36% in SLE, which is much lower than that of CVST in BD (7.8% [11]), making this condition quite rare. It is not clear when and why CVST could happen and whether this condition is due to high SLE activity.

Several mechanisms might contribute to the formation of CVST in SLE patients. Endothelial cell injury caused by immune complex-induced vasculitis was thought to play an important role in NPLE, which includes CVST. The existence of APLs may be another cause of CVST in SLE [12]. Thrombophilia is related to some SLE complications, such as nephritic syndrome [13] and hyperfibrinogenemia caused by chronic inflammatory status and could represent another etiologic factor. Infections of the middle ear, facial skin, or intracalvarium, which were known as the most common causes of CVST [14], were secondary consequences of GC and immunosuppressant treatment and are sometimes minor factors associated with CVST in SLE. Based on our data, no related craniofacial infections were found in the SLE patients complicated with CVST. Renal involvement was documented in 12 cases, but few of them fulfilled the criteria of nephritic syndrome. APLs were found much more frequently (41.2%) in this group compared to SLE patients without CVST (15.7%), and it hints that these molecules are involved in CVST etiology in SLE. Moreover, most cases had high disease activities and good responses to GC (systemic administration or intrathecal injection) and immunosuppressants, which supports that cerebral vasculitis might be another possible CVST mechanism.

Headaches, which can be persistent and severe and the only neurological sign, are the most frequent symptom of CVST and occur in about 90 percent of CVST patients [15]. In the current study, 88.2% patients suffered from headache, most of which increased gradually over days or weeks, while some had acute-onset mimicking or were accompanied by or even initially presenting as a SAH [16, 17]. Four cases (23.5%) coexisted with SAH in this cohort. The cause of headaches is likely intracranial hypertension (ICH), which can also cause vomiting, papilledema, and diplopia due to involvement of the cranial nerves. Thirteen patients (76.5%) were confirmed as ICH and the pressures in 7 cases were higher than 330 mm H_2_O in the current study. Headache is also a common manifestation of NPLE [18]. Most lupus headaches are chronic and are not accompanied by ICH or other neurological signs, while headaches in the majority of CVST cases are acute or subacute. Besides headaches, about half of patients develop other neurological signs to indicate their location to practitioners [19]. SSS involvement frequently leads to paralysis and seizure, while thrombosis of the TS can be an underlying cause of acute aphasia. Isolated or multiple cranial nerve palsies are usually caused by blockade of TS or SS. Cavernous sinus syndrome, manifesting as oculomotor nerve palsies, facial pain, sensory loss in the distribution of the trigeminal nerve, or proptosis and chemosis, is often caused by infections and is very rare in SLE.

Analysis of the cerebrospinal fluid through lumbar puncture is almost always nonspecific but is necessary to differentiate infections. In this study, slightly elevated CSF protein was found in more than half of patients (9/17, 52.9%). MRV is now the most sensitive tool for detecting CVST [19], but its specificity is based on meticulous knowledge of the anatomy and common variations of the cerebral sinus such as left transverse sinus hypoplasia or atresia could be seen in some patients [20]. Therefore, the diagnosis of CVST should be determined not only by imaging but also by clinical manifestations and CSF measures. SSS was reported to be the most frequently involved site (62.5%) and TS was the second (41.2%~44.7%) [15]. Infections always cause thrombosis of the cavernous sinus, TS, or SS. When thrombophilia exists, the SSS is frequently affected. Previously, there were no data concerning CVST associated with SLE. In the current study, TS was the most common site of thrombosis occurrence (14/17, 82.4%) followed by SS (9/17, 52.9%), SSS (6/17, 35.3%), and the inferior sagittal sinus (ISS, 1/17, 5.9%). Twelve CVST patients had been affected by more than one sinus (70.6%).

The current study provided information about the systemic involvement of SLE cases with CVST. The data represented that most cases were accompanied by renal and hematological involvement or arthritis, fever, and rash. The higher prevalence of thrombocytopenia in SLE patients with CVST might partly result from damage caused by autoantibodies and depletion of platelets when thrombosis occurs. The meaning of APLs was apparent and confirmed that these antibodies were involved in CVST in SLE. Concerning SLE activity, the SLEDAI of the CVST group was higher than the controls, indicating that the occurrence of CVST might parallel the systemic active situation of SLE.

The treatment of patients with CVST should include four aspects: removal of precipitating factors, administration of antithrombotic therapy, lowering of ICH, and relieving of neurological symptoms [15, 21, 22]. For SLE patients with CVST, the underlying cause of CVST is SLE. The current study showed that these cases had high SLE activity and rapid disease progression so that aggressive treatments to control SLE are necessary and important. In order to achieve clinical remission as soon as possible, most cases needed GC pulse therapy combined with CTX, which is one of the most powerful immunosuppressants. Anticoagulation was considered as the cornerstone of CVST treatment [9, 21]. Heparin [23] or LMWH was utilized by 11 cases and followed by warfarin in the current study, while SAH [24] and severe thrombocytopenia were contraindications to anticoagulation in other patients. Patients had also been treated with dehydration drugs and intrathecal injections of dexamethasone (10 mg) to normalize their ICP.

In the past, the mortality rate of CVST in SLE reached 30%~50% [25]. In part due to modern neuroimaging and LMWH administration, the mortality rate was reduced to 8%~14% and the outcomes continued to improve [26, 27]. In the current study, the mortality was 11.8% (2/17, 1 case died as the result of suicide rather than CVST). Complete or partial recovery was observed in most patients who survived (13/17, 76.5%), while no patient experienced a relapse of CVST or had a poor outcome with permanent neurological deficits during the mean 3-year follow-up.

## 5. Conclusions

As one of the rare and severe complications of SLE, CVST usually occurs in active SLE patients and is accompanied by systemic involvement (especially thrombocytopenia). CVST in SLE may be caused by a variety of pathogenic factors, such as vasculitis and APLs. The clinical picture of CVST is nonspecific and may vary significantly due to different venous sinus involvements, which can make the diagnosis quite difficult. Lumbar puncture and MRV are necessary for establishing a diagnosis of CVST. Treatment should include controlling SLE activity, anticoagulation administration, and ICH management. Early diagnosis and prompt management of CVST and the underlying disease could significantly improve the prognosis of lupus with CVST.

## Figures and Tables

**Table 1:** Neurologic features of the 17 patients with SLE and CVST.

Parameter	Value^*∗*^
Age at CVST diagnosis, mean ± SD years	28.4 ± 11.3
CVST as initial feature of SLE	1 (5.9%)
Type of onset	
Acute	12 (70.6%)
Progressive	5 (29.4%)
Clinical features of CVST	
Persistent headache	15 (88.2%)
Nausea/vomiting	8 (47.1%)
Conscious disturbance	7 (41.2%)
Blurred vision/diplopia	6 (35.3%)
Edema of eyelids or conjunctiva	6 (35.3%)
Seizure	6 (35.3%)
Hearing loss	2 (11.8%)
Site of CVST occlusion	
Superior sagittal sinus	6 (35.3%)
Transverse sinuses	14 (82.4%)
Sigmoid sinuses	9 (52.9%)
Inferior sagittal sinus	1 (5.9%)
Elevated CSF pressure	13 (76.5%)
Subarachnoid hemorrhage	4 (23.5%)
Sinusitis	3 (17.6%)
Cerebral ischemia or infarction	2 (11.8%)

^*∗*^Values are the number (percentage); CVST = cerebral venous sinus thrombosis; CSF = cerebrospinal fluid.

**Table 2:** Clinical manifestations of SLE patients with CVST.

Number	Gender/age (y)	Organ involvement	SLEDAI	Site of CVST occlusion	Treatment	Outcome
1	F/16	F, K, H	21	SSS, (B) TS, (B) SS	GC (pulse) + CTX	Lost
2	F/18	F, K, H, C	25	(L) TS	GC (pulse) + CTX	Died
3	F/17	F, R, A, K	22	(R) TS, (R) SS	GC + CTX	Died
4	F/20	F, H	15	SSS	GC (pulse) + CTX	Survived
5	M/38	H, K	20	SSS, (B) TS, (B) SS	GC (pulse) + CTX + FK506	Survived
6	F/36	R, A, H, C, P	17	(L) TS, (L) SS	GC + CTX	Survived
7	F/43	R, A, S	18	SSS, (L) TS	GC + CTX	Survived
8	F/52	A, K, S	19	(R) ISS, (L) TS	GC (pulse) + CTX	Survived
9	F/30	F, K, P	24	(R) SS, (R) TS	GC (pulse) + CTX	Survived
10	F/20	S, K, H, TTP	30	SSS, (L) TS	GC (pulse) + CTX + MMF + plasmapheresis	Survived
11	M/30	None	16	SSS, (B) TS	GC (pulse) + CTX	Survived
12	M/14	F, GI, K	20	(R) SS	GC (pulse) + CTX	Survived
13	F/41	R, A, K	18	(B) TS, (B) SS	GC (pulse) + CTX	Died
14	M/28	K, A	20	(L) SS, (L) TS	GC (pulse) + CTX	Survived
15	F/30	R, H	17	(R) TS	GC + CTX	Survived
16	M/34	K	20	(L) SS	GC (pulse) + CTX	Survived
17	F/15	K, CAPS	19	SSS	GC (pulse) + CTX	Survived

*Organ Involvement*. F: fever, R: rash, H: hemocytopenia, C: cardiac involvement, K: kidney disease, A: arthritis, P: pulmonary involvement, S: serositis, GI: gastrointestinal involvement, and CAPS: catastrophic antiphospholipid syndrome.

*Site of CVST Occlusion*. B: bilateral, L: left, R: right, SSS: superior sagittal sinus, ISS: inferior sagittal sinus, SS: sigmoid sinus, and TS: transverse sinus.

*Treatment*. GC: glucocorticoid, CTX: cyclophosphamide, and MMF: mycophenolate mofetil.

**Table 3:** Clinical comparison of SLE patients with CVST and without CVST.

Characteristics	SLE with CVST (*N* = 17)	SLE without CVST (*N* = 51)	*P* value
Age (years, mean ± SD)	28.4 ± 11.3	32.7 ± 11.1	0.168
Gender (F/M)	12/5	43/8	0.620
Disease duration (months, mean ± SD)	30.0 ± 39.6	38.2 ± 33.0	0.400
Fever	6/17 (35.3%)	24/51 (47.1%)	0.398
Rash	5/17 (41.2%)	21/51 (41.2%)	0.300
Musculoskeletal involvement	6/17 (35.3%)	27/51 (52.9%)	0.207
Hemocytopenia	10/17 (58.8%)	33/51 (64.7%)	0.516
Lymphopenia	4/17 (23.5%)	18/51 (35.2%)	0.320
Anemia	9/17 (52.9%)	19/51 (37.3%)	0.255
Thrombocytopenia	10/17 (58.8%)	12/51 (23.5%)	0.007^*∗*^
Serositis	5/17 (29.4%)	11/51 (21.6%)	0.523
Kidney involvement	12/17 (70.6%)	30/51 (58.8%)	0.387
Gastrointestinal involvement	1/17 (5.9%)	7/51 (13.7%)	0.669
Other neurological manifestations	6/17 (35.3%)	11/51 (21.6%)	0.334
Cardiovascular involvement	2/17 (11.8%)	7/51 (13.7%)	1.000
Serum albumin (g/L, mean ± SD)	29.8 ± 6.9	30.7 ± 8.6	0.715
ESR (mm/1 h, mean ± SD)	42.2 ± 24.6	38.0 ± 24.5	0.537
Hypocomplementemia	12/17 (70.6%)	37/51 (72.5%)	1.000
Anti-dsDNA	8/17 (47.1%)	35/51 (68.6%)	0.110
Anti-Sm	6/17 (35.3%)	19/51 (37.3%)	0.885
Anti-RNP	5/17 (29.4%)	24/51 (47.1%)	0.203
Anti-SSA	12/17 (70.6%)	28/51 (54.9%)	0.198
Anti-SSB	1/17 (5.9%)	6/51 (11.8%)	0.670
Anti-rRNP	3/17 (17.6%)	12/51 (23.5%)	0.745
APL	7/17 (41.2%)	8/51 (15.7%)	0.043^*∗*^
SLEDAI (CVST included, mean ± SD)	20.1 ± 3.7	12.8 ± 5.3	0.001^*∗*^

^*∗*^ *P* < 0.05.

Anti-dsDNA: antidouble stranded DNA antibody, Anti-Sm: anti-Smith antibody, Anti-SSA: anti-SSA antibody, Anti-SSB: anti-SSB antibody, Anti-RNP: anti-u1 small-nuclear RNA-protein antibody, Anti-rRNP: antiribosomal RNA-protein antibody, APL: antiphospholipid antibody, and SLEDAI: SLE disease activity index.
